# Dendrimer-based Nanoparticle for Dye Sensitized Solar Cells with Improved Efficiency

**DOI:** 10.4172/2157-7439.1000496

**Published:** 2018-04-13

**Authors:** William Ghann, Hyeonggon Kang, Jamal Uddin, Sunalee J Gonawala, Sheikh Mahatabuddin, Meser M Ali

**Affiliations:** 1 Center for Nanotechnology, Department of Natural Sciences, Coppin State University, Baltimore, MD21216, USA; 2 Department of Neurosurgery, Cellular and Molecular Imaging Laboratory, Henry Ford Hospital, Detroit, MI, USA

**Keywords:** Nanoparticle, Solar cells, Dendrimer, Dye

## Abstract

Dye sensitized solar cells were fabricated with DyLight680 (DL680) dye and its corresponding europium conjugated dendrimer, DL680-Eu-G5PAMAM, to study the effect of europium on the current and voltage characteristics of the DL680 dye sensitized solar cell. The dye samples were characterized by using Absorption Spectroscopy, Emission Spectroscopy, Fluorescence lifetime and Fourier Transform Infrared measurements. Transmission electron microscopy imaging was carried out on the DL680-Eu-G5PAMAM dye and DL680-Eu-G5PAMAM dye sensitized titanium dioxide nanoparticles to analyze the size of the dye molecules and examine the interaction of the dye with titanium dioxide nanoparticles. The DL680-Eu-G5PAMAM dye sensitized solar cells demonstrated an enhanced solar-to-electric energy conversion of 0.32% under full light illumination (100 mWcm^−2^, AM 1.5 Global) in comparison with that of DL680 dye sensitized cells which recorded an average solar-to-electric energy conversion of only 0.19%. The improvement of the efficiency could be due to the presence of the europium that enhances the propensity of dye to absorb sunlight.

## Introduction

Dye sensitized solar cells (DSSCs) are a promising renewable form of energy that are easy to fabricate, portable, low cost, environmentally friendly and have a relatively high solar-to-electric energy conversion efficiency [1–5]. Dye sensitzed solar cells were invented by Michael Gratzel et al. in 1991 and has since generated a lot of interest leading to an exponential growth in reseach relating to DSSC [6,7]. It is generally composed of two conductive glass electrode, a photoande and a counter electrode, with a redox electrolyte sandwiched between them. The electrolyte ensures the regeneration of charge. Much of the work in the field has centered on the manipulation and enhancement of the afformentioned components and the resulting effect on the production of highly efficient solar cells [8–11]. The photoanode comprises of a dye sensitized titanium dioxide nanoparticle film on a fluorinedoped tin oxide (FTO) transparent glass. The dye adsorbed on the titanium dioxide semiconductor, absorbs radiant energy and generate charges which travel through the semiconductor to the conductive layer and subsequently through an external ciruit to the cathode. The electrolyte commonly used in the fabrication of dye sensitized solar cell is Iodine/triodine redox couple system. Other electrolyte have been developed which have also been found to enhance the efficiency of solar cells [12,13]. However, the most critical component of the device is the sensitizing dye that is utilized in the construction of the solar cell. The dye is expected to show a high absorption in the UV-visible and infrared region. The dye must also have anchoring groups, usually carboxylic groups capable of attaching to the surface of the titanium dioxide nanoparticles [14]. A number of different dyes have been explored in the production of dye sensitized solar cells [15–17]. DyLight 680 (DL680) is a near infrared fluorescent dye that is frequently used in bioanalysis and bioimaging. It is often conjugated to other imaging probes to afford multimodal imaging. DL680 conjugated with europium has been successfullly employed for MRI and fluorescene imaging [18–20]. This dye has the potential for use as sensitizing dye in dye sensitized solar cells due to the presence of large number of surface carboxylic groups capable of binding to the titanium dioxide semiconducter, consequently enhancing the transport of charge. In this study, DL680 conjugated to an europium incorporated generation 5 poly(amidoamine) (G5PAMAM) denderimer (DL680-Eu-G5PAMAM) was deployed as a sensitizing dye for the fabrication of solar cells in order to compare its perfomance witht that of bare DL680 dye sensitized solar cells.

## Experimental Section

### Materials

Titanium dioxide powder (Degussa P25) was purchased from the institute of chemical education. Fluorine tin oxide (FTO) conducting glass slides were purchased from Harford glass company, Hartford City, Indiana. Sodium Hydroxide (NaOH), acetone (C_3_H_6_O), ethanol (C_2_H_5_OH), and acetic acid (CH_3_COOH) were purchased from Sigma- Aldrich and were used without further purification. Graphite used in making cathode slides was purchased from TED PELLA, INC.

The photoanode was prepared on a fluorine-doped SnO_2_ (FTO) conducting glass substrate. The FTO glass slides were cleaned with detergent solution, rinsed first with water, and then with ethanol. The FTO glass substrates were subsequently spin coated with TiO_2_ paste prepared from TiO_2_ powder, acetic acid, and soap water. The TiO_2_ coated FTO slides were annealed at 450 °C for an hour and allowed to cool to room temperature. Using Field Emission Scanning Electron Microscopy cross-sectional imaging, the thickness of the TiO_2_ layer was determined to be approximately 8 µm. To prepare the cathode, graphite paint was spread uniformly on the cleaned FTO glass and allowed to dry at room temperature.

### Synthesis of DL680-Eu-G5PAMAM

The synthesis of DL680-Eu-G5PAMAM was conducted by following the method described in our previous report [18]. Briefly, Eu-DOTA-Gly4 was synthesized first [18], and then was coupled with NHS and 1-ethyl-3-(3-dimethylaminopropyl) carbodiimide. HCl in 2-(N-morpholino)ethanesulfonic acid buffer. The resulting active ester, Eu-DOTA-Gly4-NHS, was added in aliquots of a G5 PAMAM dendrimer and then allowed to stir at room temperature for 24 h. The solution was filtered using a centrifugal filter unit with a 10,000-molecular weight cut-off (Millipore Inc., MA, USA). Finally, the solution was lyophilized to obtain [Eu-DOTA-Gly4]42–G5PAMAM] as white solid. Then, a solution of DL680-NHS ester (50 mg, ~0.071 mmol; Thermo Fisher Scientific, IL, USA) in dimethyl sulfoxide was added to a stirred solution of Eu-G5PAMAM (200 mg, 0.071 mmol) in 2 ml of PBS, and the reaction was stirred at room temperature for 24 h. The reaction mixture was diafiltrated using Amicon Ultra centrifugal filter unit with a 10,000-molecular weight cut-off. The solution was lyophilized to obtain 210 mg of solid (~066 mmol, 93% yield).

### Fluorescence lifetime measurements

DL680-Eu-G5PAMAM conjugate and DL680 dye were each dissolved in 3 mL of ethanol for fluorescence lifetime measurements. To prevent inner filter effect, absorption measurements were first carried out to ensure the absorbance of the dyes was less or equal to 0.15 a.u. Fluorescence decays were measured using Horiba Deltaflex fluorescence lifetime system using the time-correlated single-photon counting (TCSPC) technique with the PPD-850 picosecond photon detection module. The excitation source was 532 nm light-emitting diodes (Delta LED) with 532 nm.

### Fabrication of solar cell

The DSSCs were prepared according to previously published protocols [21–23]. The TiO_2_ coated FTO glass was immersed in freshly prepared solutions of DL680-Eu-G5PAMAM and DL680 for a period of two hours. The counter electrode (cathode) was prepared by painting colloidal graphite on FTO glass substrate. Each of DL680-Eu-G5PAMAM nanoparticles and DL680 dye-sensitized electrodes and their respective counter electrode were assembled to form solar cells sandwiched with a redox (I^−^/I^3−^) electrolyte solution.

### Characterization techniques

Steady-state absorption spectra of DL680-Eu-G5PAMAM and DL680 dye solutions were acquired using UV-3600 Plus from Shimadzu. Steady-state fluorescence spectra were recorded on the fluorescence Nanolog Spectrofluorometer System from Horiba Scientific (FL3-22 iHR, Nanolog). ATR spectra was obtained with a Thermo Nicolet iS50 FTIR. The morphology of each film was analyzed using field emission scanning electron microscopy (FESEM; JSM-7100FA JEOL USA, Inc.). Transmission Electron Microscopy (TEM) images were acquired on JEM-1400 Plus (JEOL USA, Peabody, Massachusetts). The images were viewed using Digital Micrograph software from Gatan (Gatan, Inc, Pleasanton, CA). TiO_2_ paste was printed on FTO glass using WS-650 Series Spin Processor from Laurell Technologies Corporation.

### Photovoltaic properties measurement

The energy efficiencies of the fabricated DL680-Eu-G5PAMAM and DL680 DSSCs were measured using 150 W fully reflective solar simulator with a standard illumination with air-mass 1.5 global filter (AM 1.5 G) having an irradiance corresponding to 1 sun (100 mW/cm^2^) purchased from Sciencetech Inc., London, Ontario, Canada and Reference 600 Potentiostat/Galvanostat/ZRA from Gamry Instruments (734 Louis Drive, Warminster, PA 18974). The tested solar cells were masked to an area of 5 cm^2^. Each cell performance value was taken as the average of three independent samples. The solar energy to electricity conversion efficiency (η) was calculated based on the equation, η=FF × Isc × Voc, where FF is the fill factor, Isc is the short-circuit photocurrent density (mA cm^−2^), and Voc is the open-circuit voltage (V) as listed in Table 1.

## Results and Discussion

**Figure 1:**
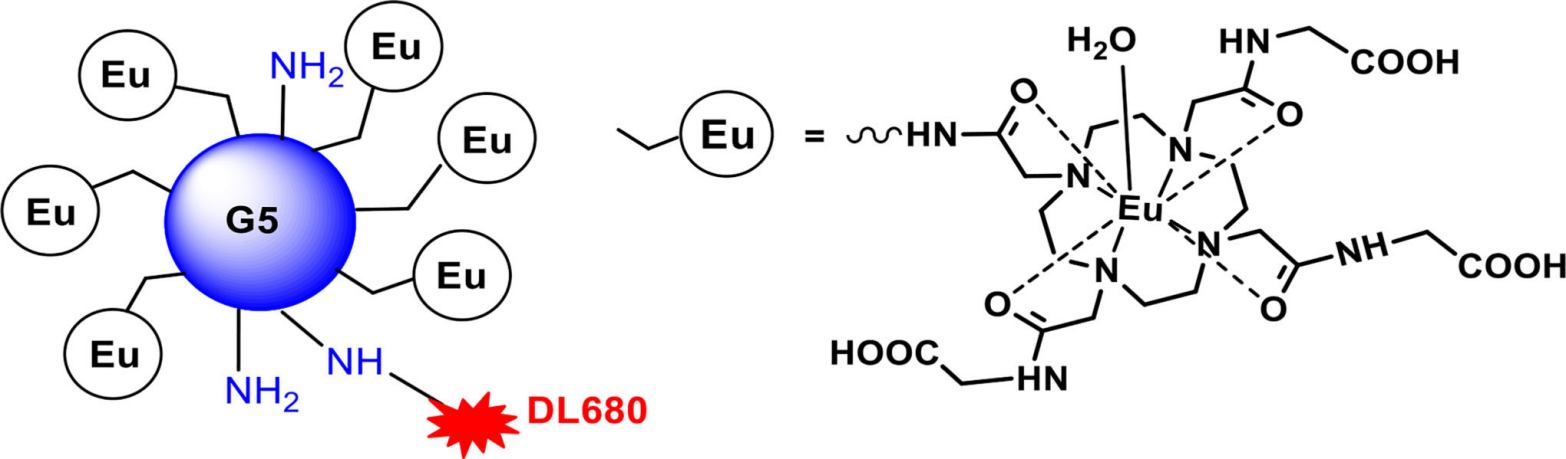
Schematic view of (Eu-DOTA-Gly4)42-G5-DL680). Eu-DOTA-Gly4 and DyLight680 (DL680) were conjugated on the amines surface of a G5 PAMAM dendrimer.

The synthesis of DL680-Eu-G5PAMAM was carried out according to a previously published method [18]. The europium compound was first synthesized and subsequently conjugated to the generation five PAMAM dendrimer via the amino groups on the surface of the dendrimer. The DL680 molecules were also conjugated on the amines surface of the dendrimer as displayed in Figure 1.

### UV-Vis absorption studies

**Figure 2:**
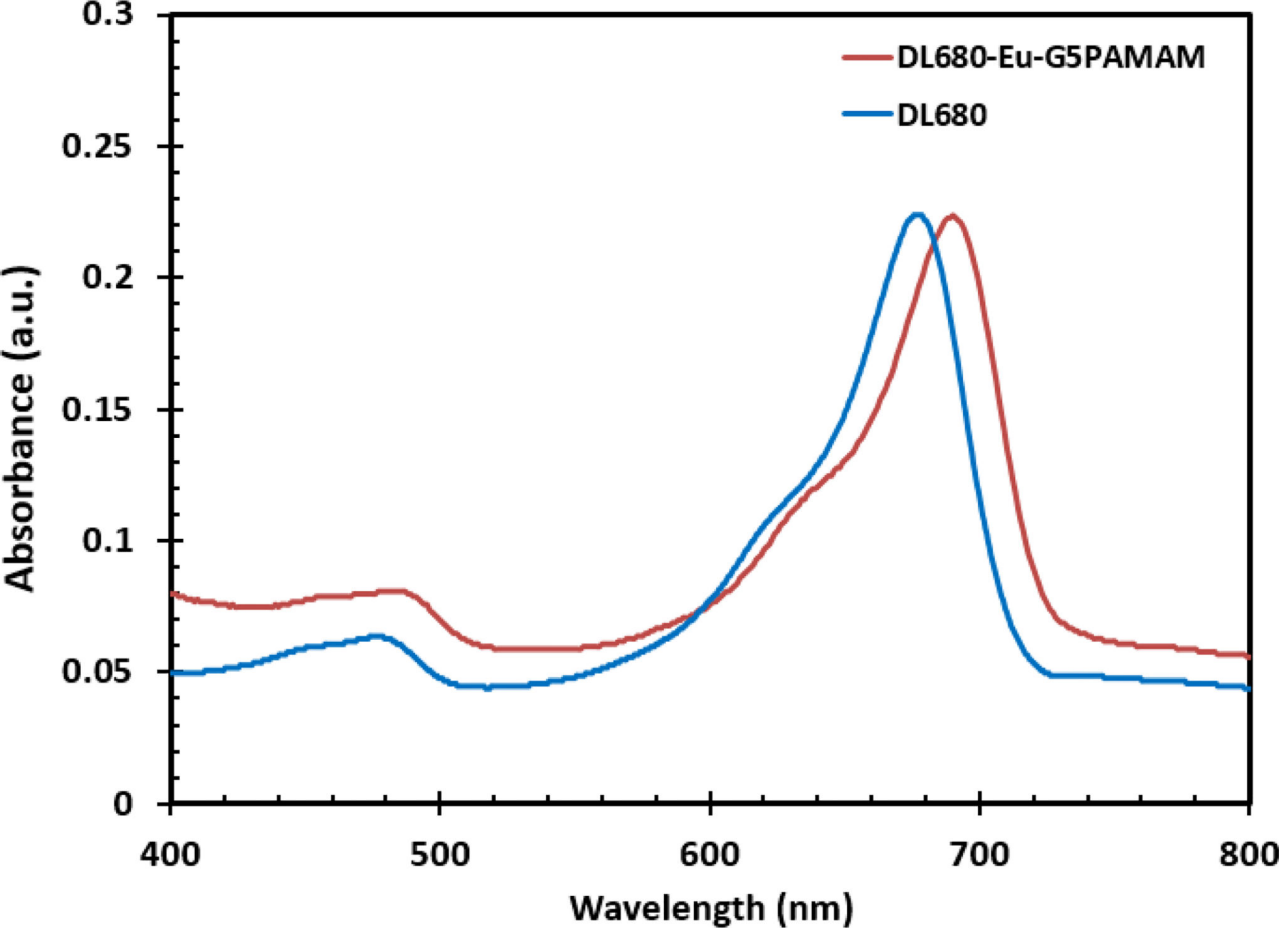
Absorption spectra of aqueous solutions of DL680 and DL680-Eu-G5PAMAM dyes.

The absorption characteristics of the DL680 and DL680-Eu-G5PAMAM dyes were studied via UV-Visible measurements. Absorption spectra of DL680 and DL680-Eu-G5PAMAM as displayed in Figure 2 show a wavelength of maximum absorption of DL680 to be 677 nm and that of DL680-Eu-G5PAMAM to be 690 nm. Therefore, conjugation DL680 with Eu-G5PAMAM resulted a red shift from 677 nm to 690 nm. This is an indication of the successful conjugation of the DL680 dye to the europium complex. In addition to the main absorption band that extends to the near infrared region, there is another band which appears at 485 nm. Thus, absorption encompasses most of the visible spectral range and enters the near-infrared region. It is also observed that the light absorption of DL680-Eu-G5PAMAM in the visible region is stronger than that of DL680 which suggest that it would be a better sensitizing agent.

### Steady state fluorescence studies

**Figure 3:**
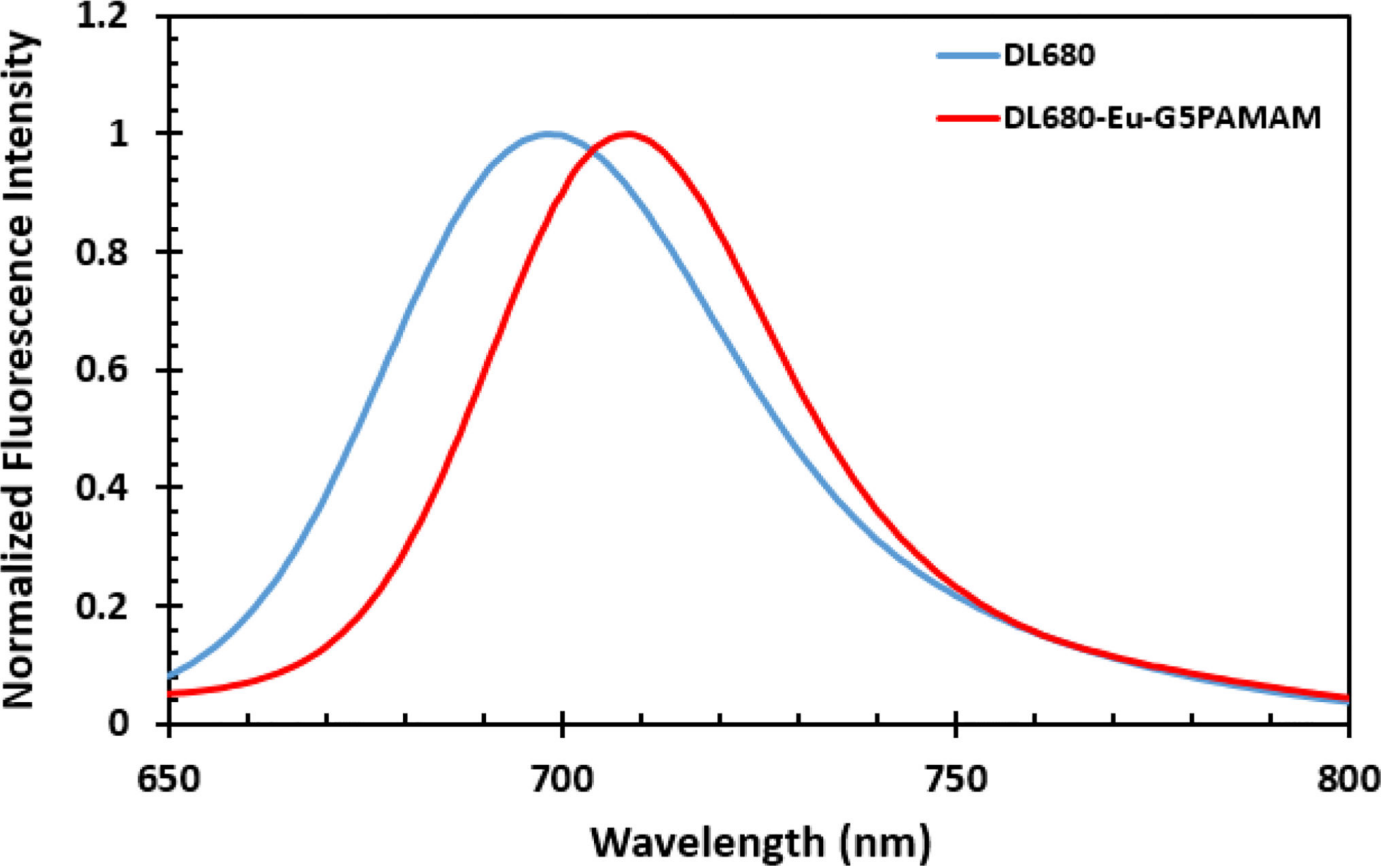
Emission spectra of DL680 and DL680-Eu-G5PAMAM in water.

The steady state fluorescence spectra of DL680 and DL680-Eu-G5PAMAM were taken as part of the photophysical studies on the dyes and are shown in Figure 3. The measurements were carried out in water with an exciting light of 600 nm. The emission maximum of DL680-Eu-G5PAMAM at 709 nm is redshifted with respect to DL680 which exhibited an emission maximum at 698 nm.

### IR spectroscopy analysis

**Figure 4:**
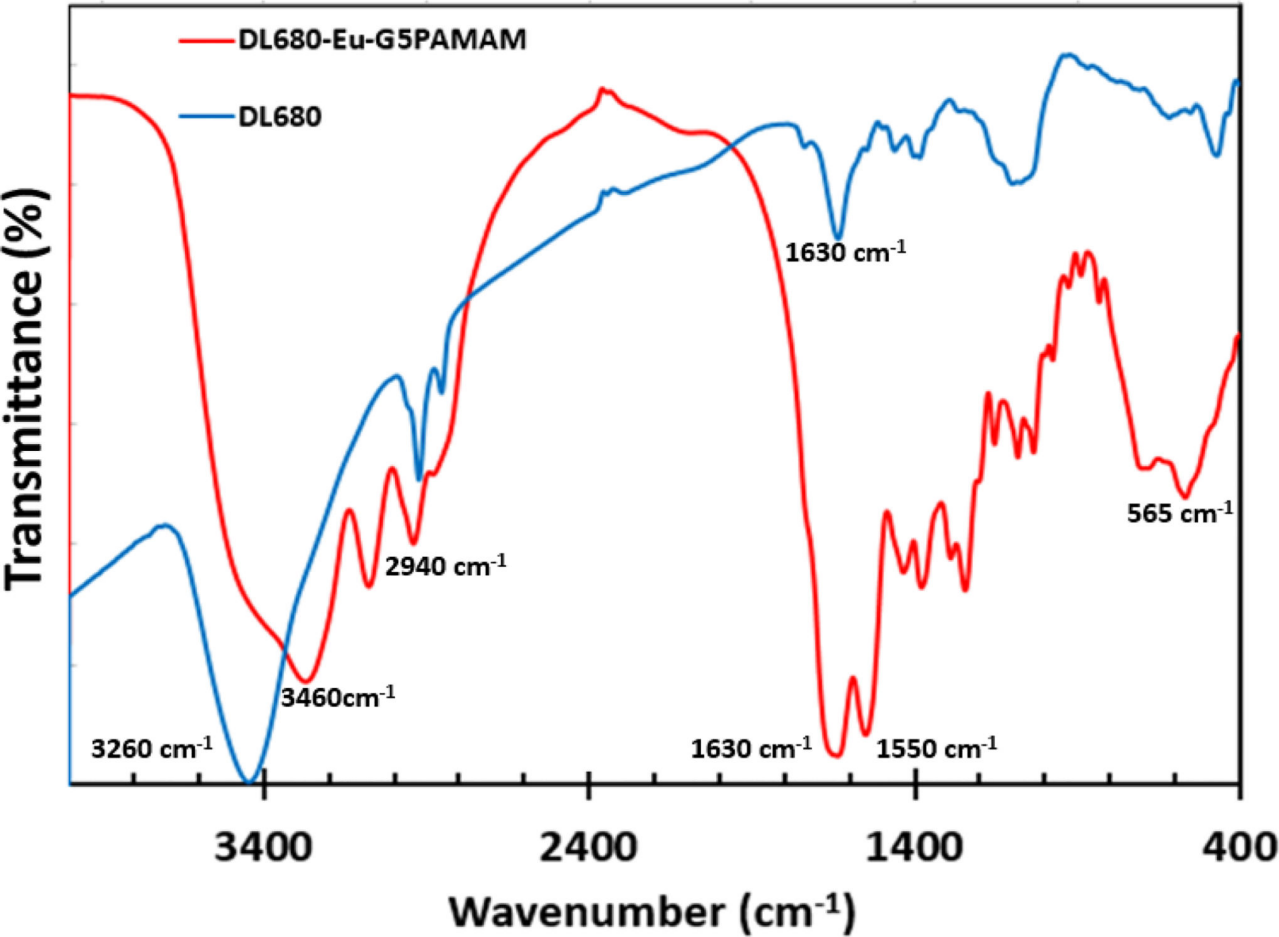
Fourier-transform infrared spectra of DL680 and DL680-Eu-G5PAMAM showing peaks of interest.

The Fourier transformed infrared spectra of the DL680 and DL680-Eu-G5PAMAM were also collected as part of the study on the photophysical properties of the dyes. The FTIR spectra of the dyes as displayed in Figure 4 verify that the DL680 dye was successfully conjugated to the Eu-G5PAMAM nanoparticle. As shown in prior studies, PAMAM dendrimer exhibits peaks at 3286 and 3345 cm^−1^ corresponding to the primary amino groups on the surface of the dendrimer [24,25]. These peaks, however, merge into a broad absorption peak upon conjugation as displayed in Figure 4. The characteristic absorption of the amide bonds also got red shifted to 1630 cm^−1^ and 1550 cm^−1^ as previously reported [24,25].

### Fluorescence lifetime studies

**Figure 5:**
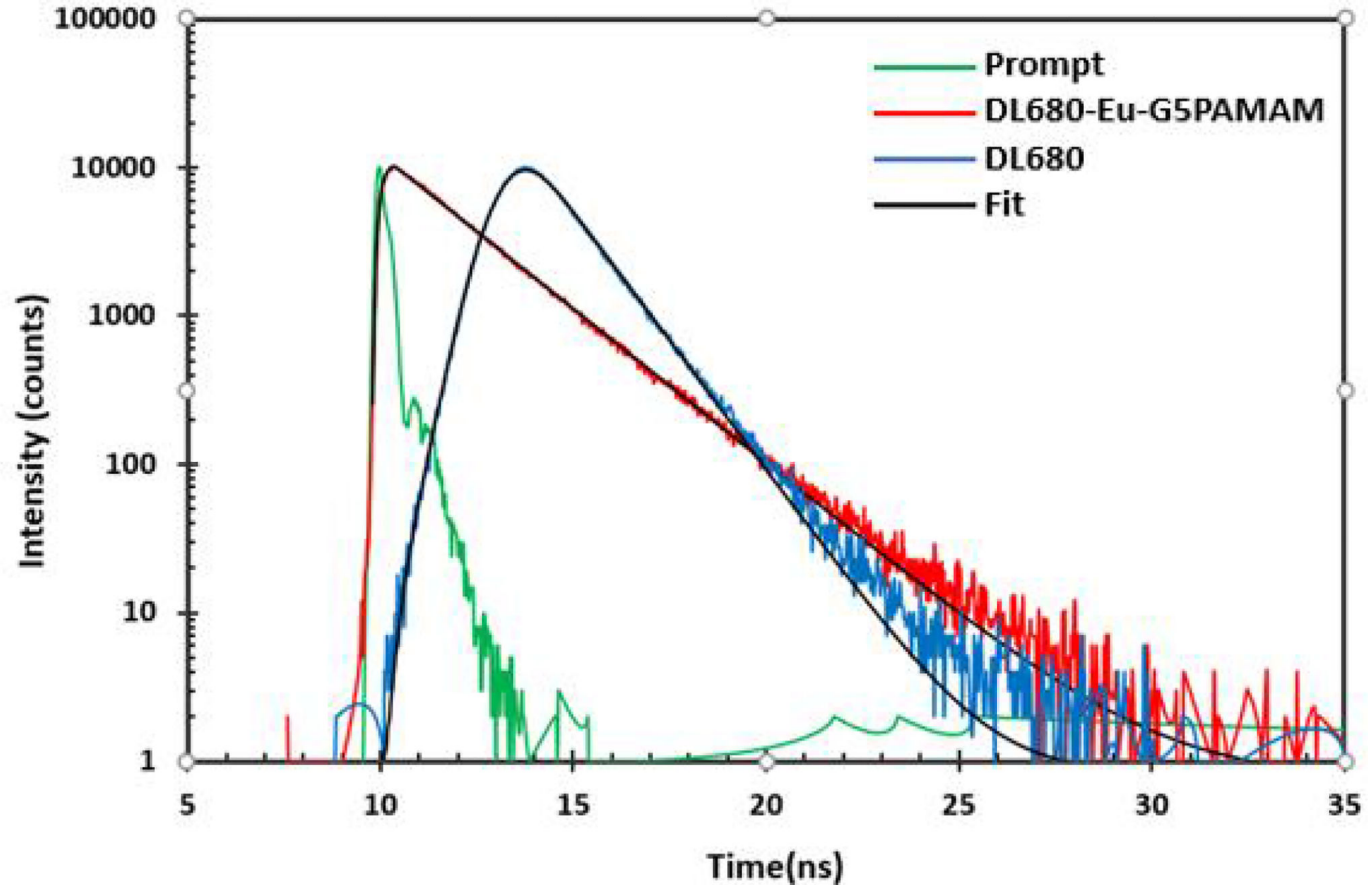
Fluorescence Lifetime measurement of DL680 and DL680-Eu-G5PAMAM.

Fluorescence lifetime (FLT) measurements (Figure 5) were carried out to study the period dye molecules reside in the excited state and how this lifetime influences the various parameters associated with DSSCs constructed with them. The lifetime of the dendritic dye was found to be 2.11 ns with a standard deviation of 0.0027 ns and that of only DL680 dye was 1.25 ns with a standard deviation of 0.0025 ns as displayed in Table 2. Luminescent europium complexes have been reported to exhibit long lifetime and are also highly stable in terms of ligand-metal dissociation and have widely been used as donors in Forster resonance energy transfers [26–28]. It is therefore reasonable that upon conjugation of the europium chelate to the DL680, the luminescence lifetime is significantly extended.

### Raman spectroscopy

**Figure 6:**
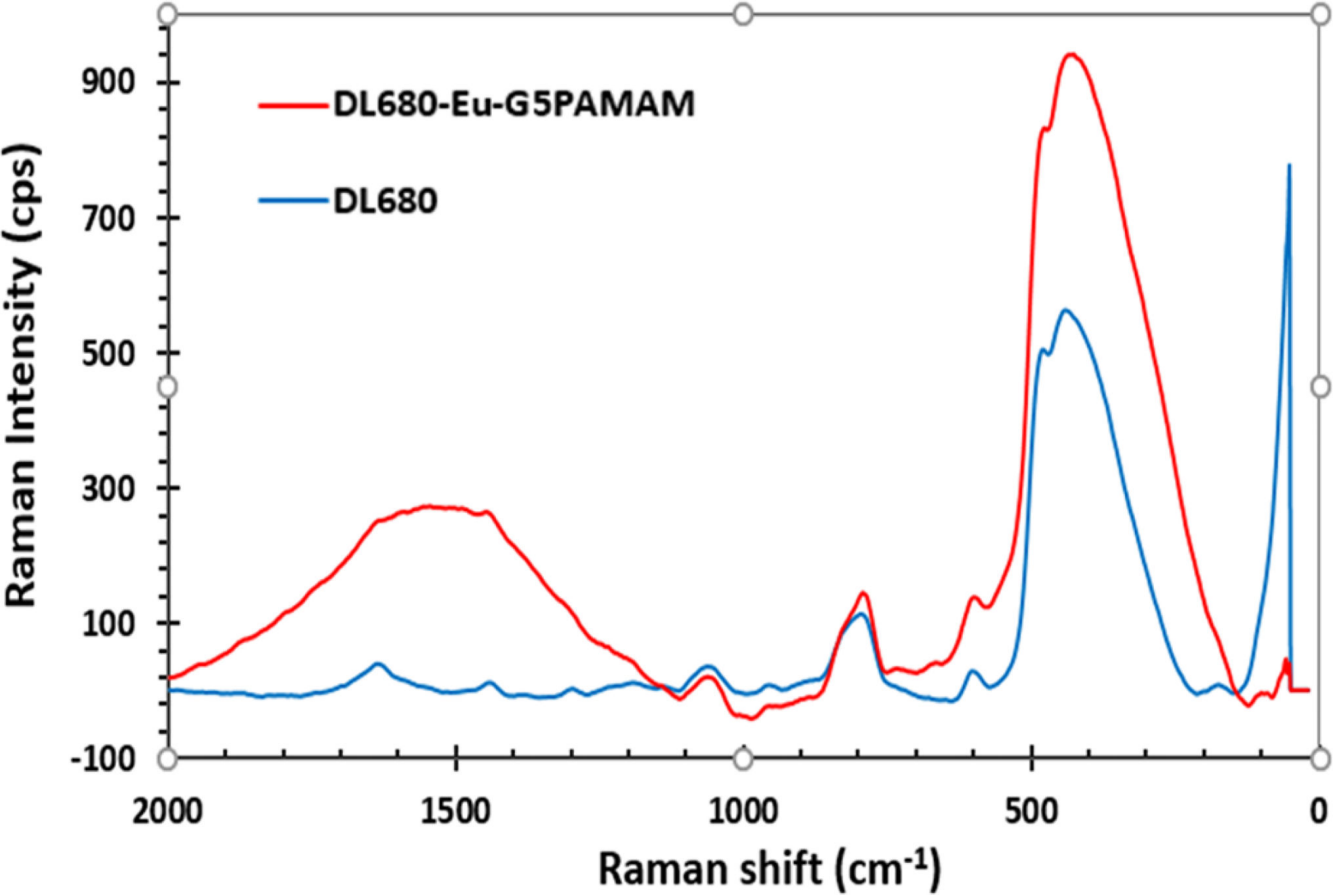
Raman Spectra of DL680 and DL680-Eu-G5PAMAM.

The DL680 and DL680-Eu-G5PAMAM were further characterized using Raman Spectroscopy. The Raman studies were performed in the range of 0–2000 cm^−1^ and the results are shown in Figure 6. There are three unique bands at 624 cm^−1^, 806 cm^−1^ and 1070 cm^−1^ associated with both DL680 and G5EuDyl680. However, there are no corresponding bands of DL680-Eu-G5PAMAM to the peaks at 1630 cm^−1^, 1430 cm^−1^ and 1290 cm^−1^ observed in the spectra of the DL680 dye. Instead a broad band that stretches 1750 cm^−1^ to 1300 cm^−1^ is observed. This broad band could correspond to the emission peak of the DL680 complex which is expected to be enhanced in the presence of the europium metal. Metal enhanced fluorescence is thus seen to occur here.

### Transmission electron microscopy imaging

**Figure 7:**
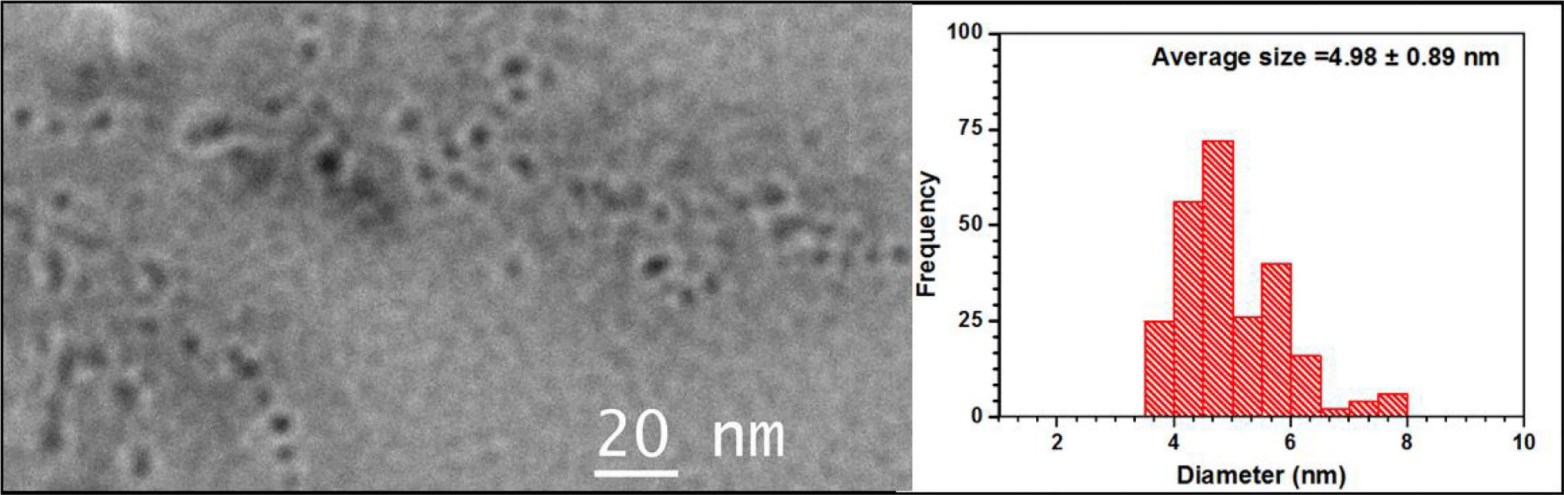
High-resolution TEM images of DL680-Eu-G5PAMAM and the corresponding histogram showing the size of the dye to be 4.98 nm with a standard deviation of 0.89.

**Figure 8:**
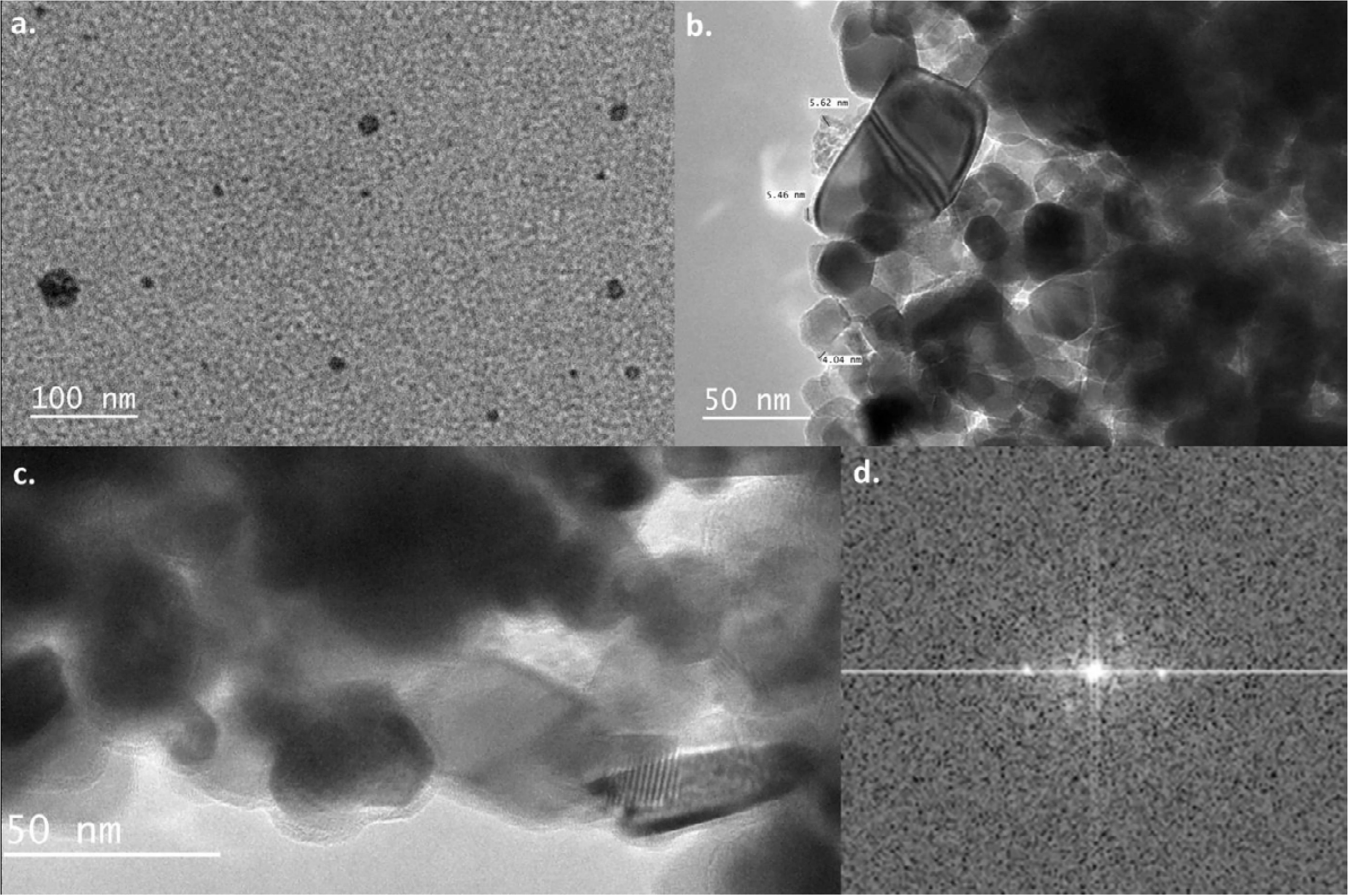
High-resolution TEM images of a TiO_2_ and a DL680-Eu-G5PAMAM /TiO_2_ samples and a FTT image of the TiO_2_: (a) high resolution TEM images of DL680-Eu-G5PAMAM dye, (b) TEM of DL680-Eu-G5PAMAM dye/TiO_2_, (c) bare TiO_2_ nanoparticles, (d) FTT Image.

The Transmission Electron Microscopy (TEM) imaging studies were carried to analyze the sizes of DL680-Eu-G5PAMAM nanoparticles and their interaction with titanium dioxide nanoparticles. Figure 7 shows the High-resolution TEM images of DL680-Eu-G5PAMAM and the corresponding histogram. The average size of the DL680-Eu-G5PAMAM nanoparticles was determined to be 4.89 nm with a standard deviation of 0.89 nm. Just a cursory look at the image as illustrated in Figure 7 shows the DL680-Eu-G5PAMAM nanoparticles to be uniformly distributed. Figure 8 displays the High-resolution TEM images of DL680-Eu-G5PAMAM (a), DL680-Eu-G5PAMAM/TiO_2_ sample (b), bare TiO_2_ nanoparticles (c), and a FTT image of the TiO_2_ (d). The images of DL680-Eu-G5PAMAM with TiO_2_ suggest considerable interaction between the dye and the TiO_2_ nanoparticles.

### Energy dispersive X-ray spectrometry studies

**Figure 9:**
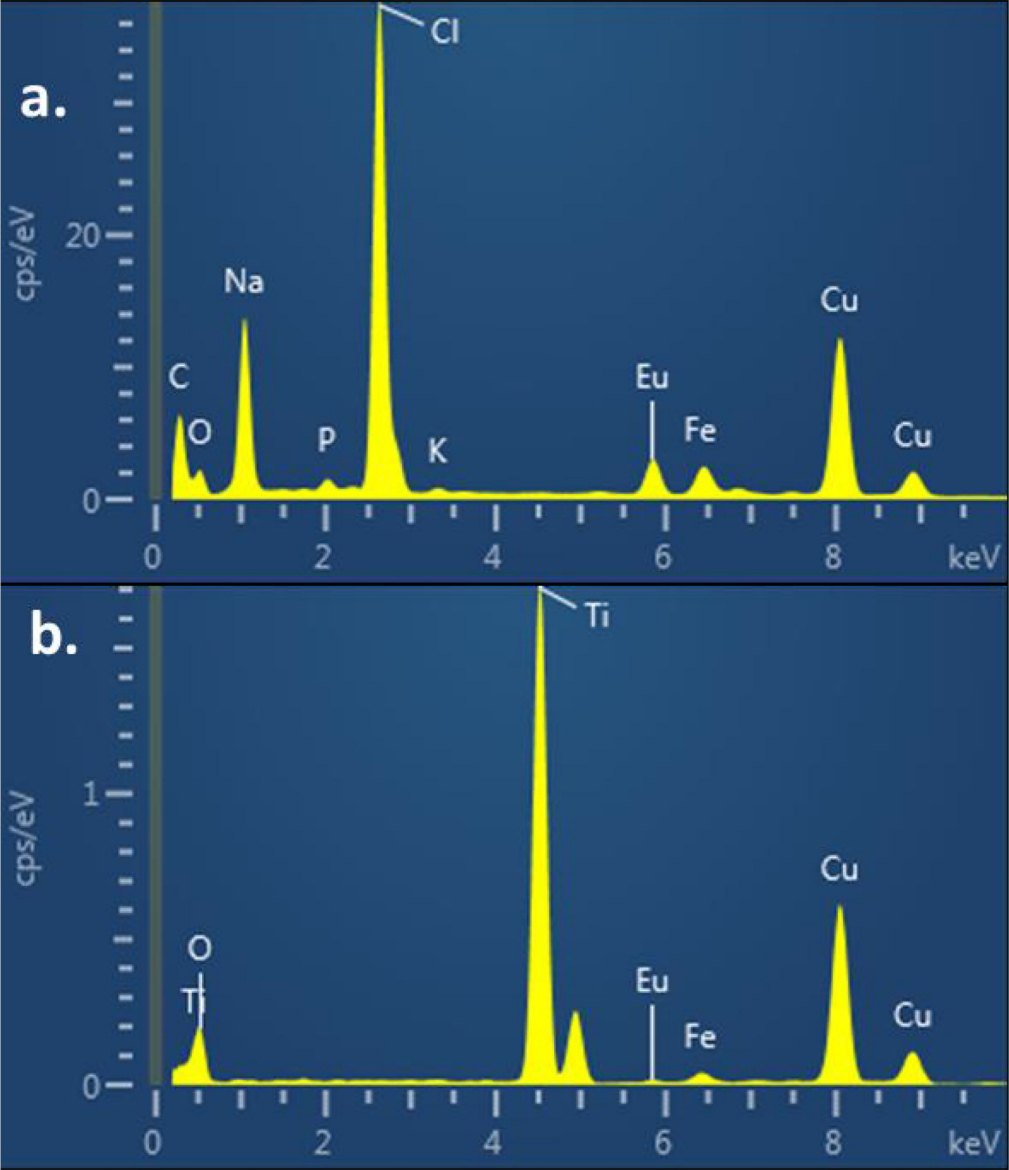
EDS Analysis of DL680-Eu-G5PAMAM without TiO_2_ nanoparticles (a) and with TiO_2_ nanoparticles (b).

The elemental composition of the dye sensitized titanium dioxide film was confirmed by Energy Dispersive X-Ray spectrometry (EDS) studies. Figure 9a shows the EDS spectra of europium conjugated dye whereas Figure 9b shows the EDS of dye sensitized titanium dioxide film. The elements carbon, oxygen, copper and europium are displayed in the EDS spectra of the DL680-Eu-G5PAMAM. The copper originates from the grid used in the measurement whereas Eu peaks confirms the presence of the dye

### Current-voltage characteristics of DL680 and DL680-Eu-G5PAMAM DSSC

**Figure 10:**
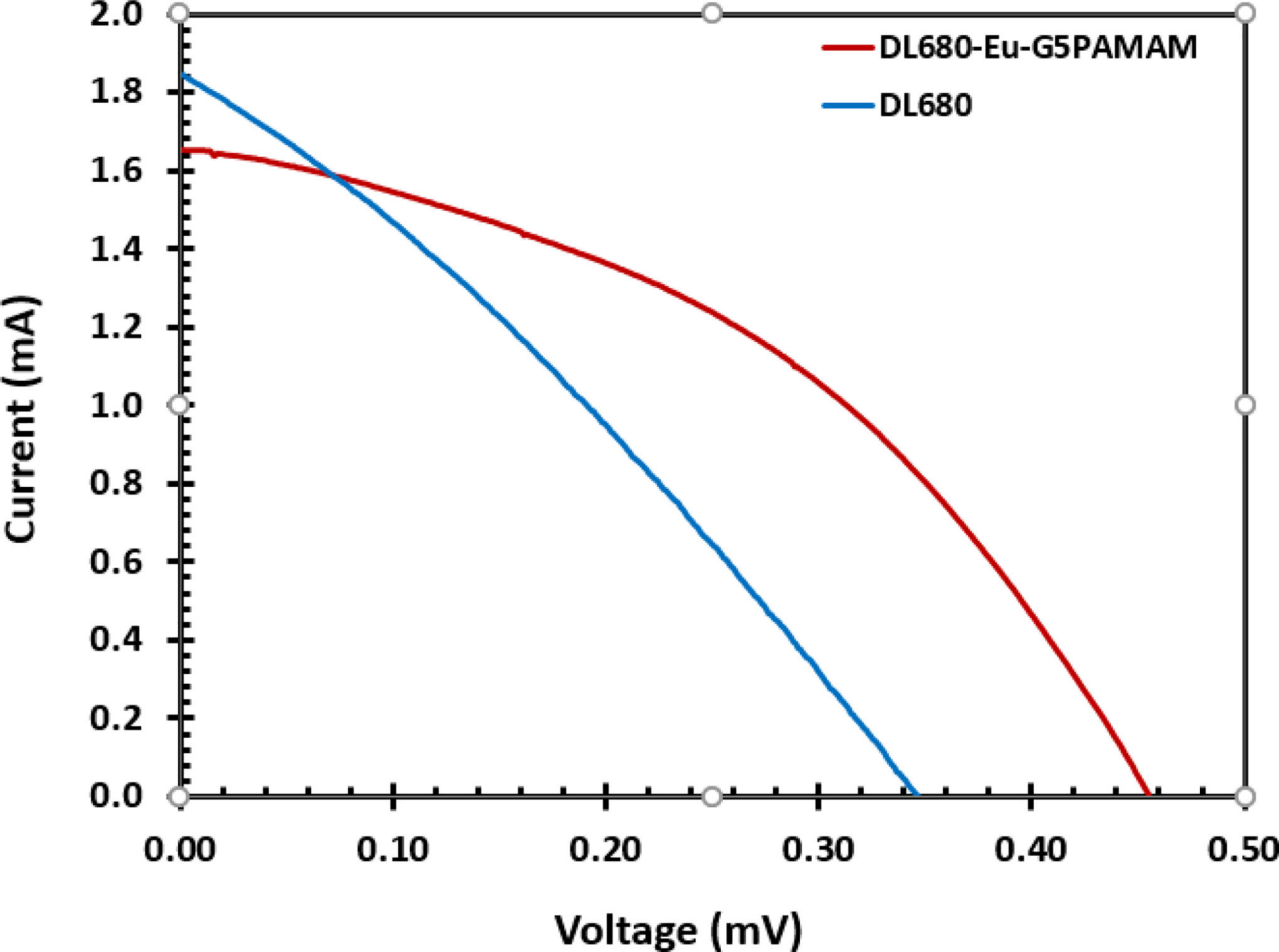
Photocurrent-voltage characteristics of DL680 and DL680-Eu-G5PAMAM dye sensitized solar cell measured under illumination of 100 mW/cm_2_ (1.5 AM).

The studies on the photovoltaic performance of DL680 and DL680-Eu-G5PAMAM DSSC under simulated solar irradiation of AM 1.5 G were undertaken and the results are displayed in Figure 10 with the corresponding current-voltage characteristics parameters presented in Table 1. In the case of DL680-Eu-G5PAMAM DSSC, 0.32% solar-to-electric conversion efficiency was realized with a short circuit current of 1.66 mA/cm^2^, open circuit voltage of 0.46 V and a fill factor of 0.42. The efficiency of the DL680 on the other hand was 0.19% which is lower than that of DL680-Eu-G5PAMAM DSSC indicating that the inclusion of europium metal improved the efficiency of the resulting device. The improvement of the efficiency could be due to the presence of the europium that enhance the propensity of dye to absorb sunlight

### Electrochemical impedance spectroscopy

**Figure 11:**
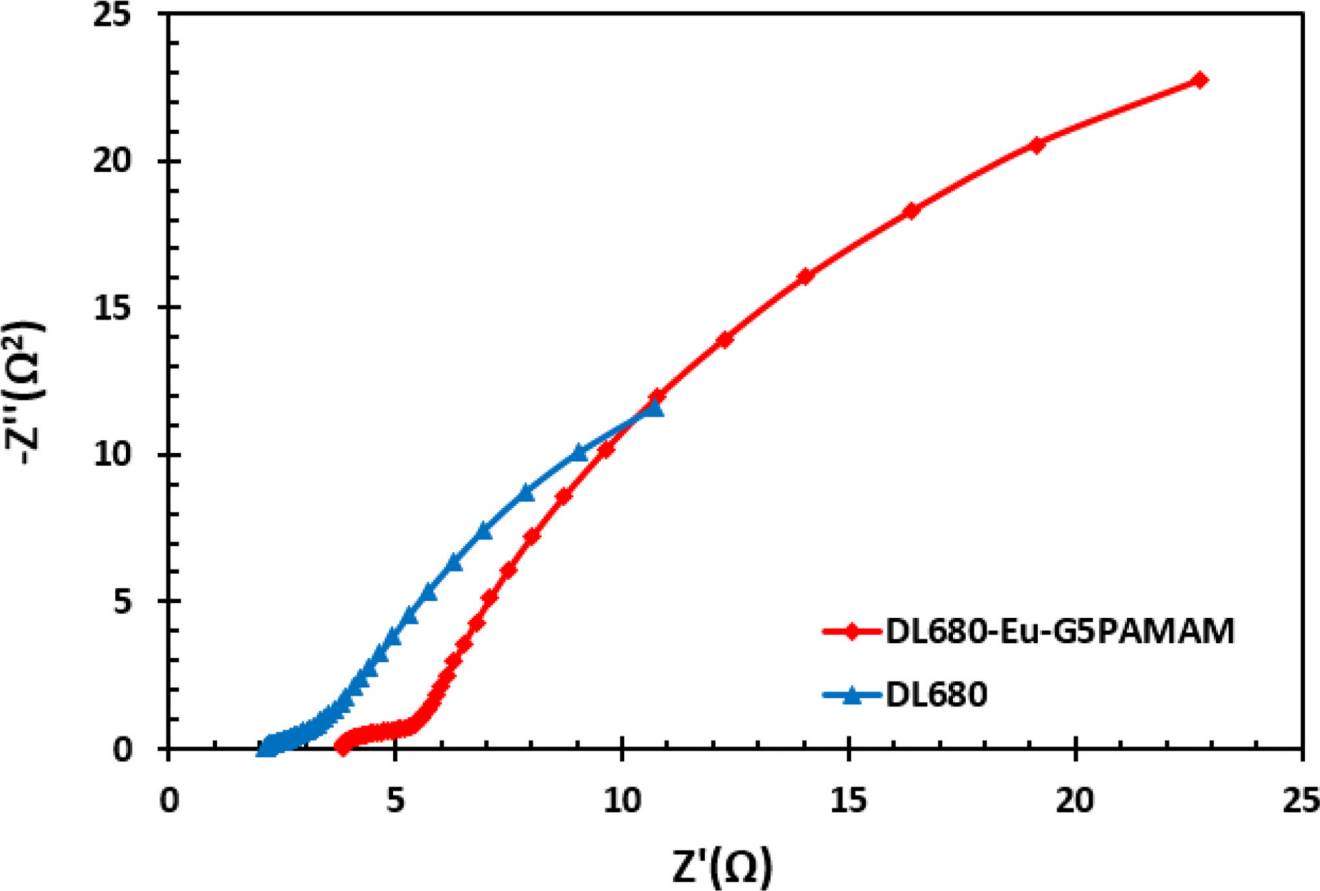
Nyquist plots for the fabricated DL680 and DL680-Eu-G5PAMAM dye sensitized solar cells showing differences in the resistances to charge transfer.

**Figure 12:**
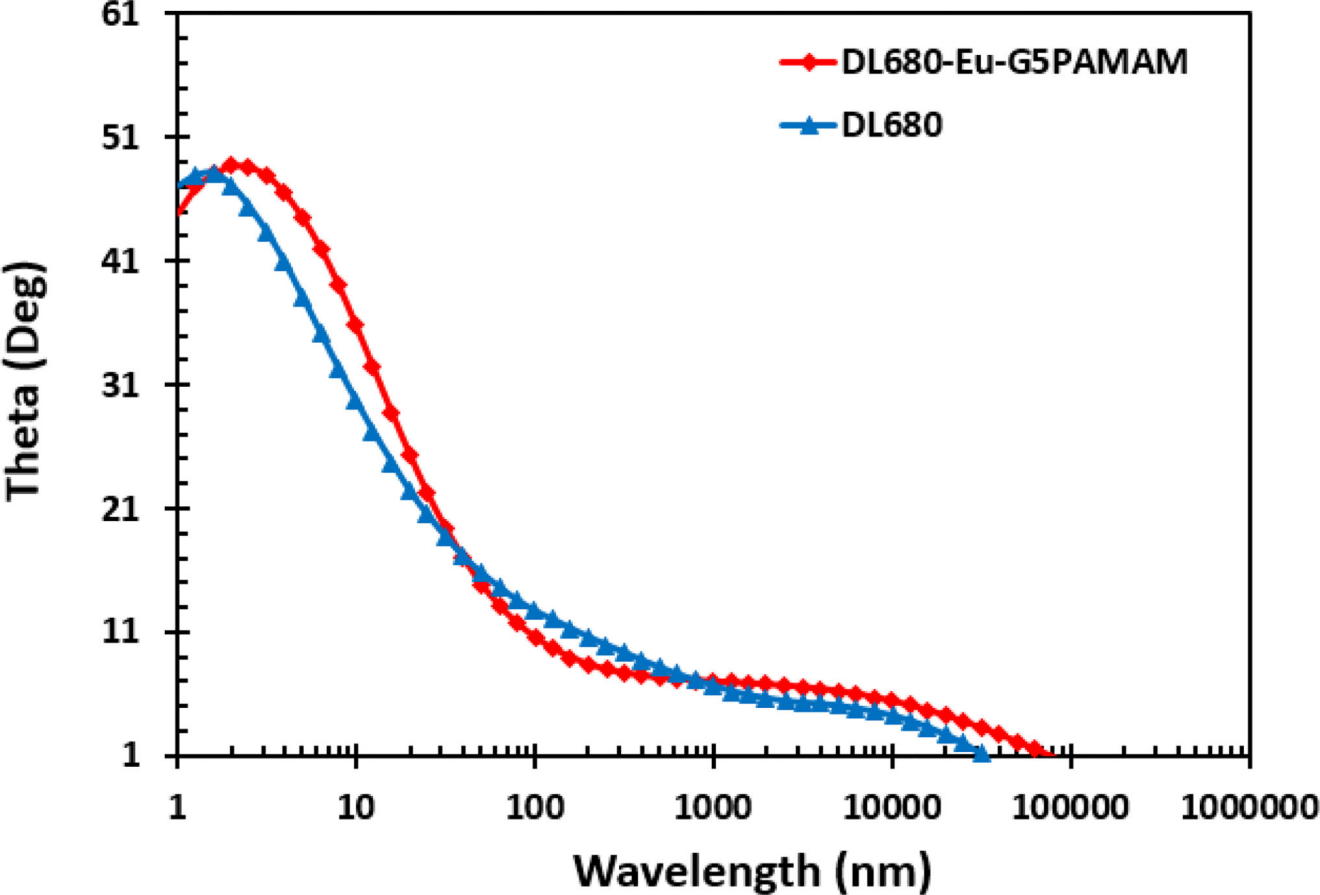
Bode plots for the fabricated DL680 and DL680-Eu-G5PAMAM dye sensitized solar cells.

Electrochemical impedance spectroscopy (EIS) is one of the vital tools employed to elucidate the charge transfer and transport processes in dye-sensitized solar cell (DSSC) devices [29–32]. EIS measurements were carried out to study the interfacial charge transfer occurring within the assembled DL680 and DL680-Eu-G5PAMAM DSSC. The results of this study are presented in the form of a Nyquist plot (Figure 11) and a Bode plot (Figure 12). The measurements were undertaken in the frequency range of 0.01 Hz to 100 KHz. The Nyquist plot shows two semi-circles with a semi-circle at the higher frequency demonstrating the electron transfer processes between the dye sensitized photoanode and the electrolyte interface. In addition, there is a little bit of difference at the intercept of the Nyquist plots of the fabricated DL680 and DL680-Eu-G5PAMAM DSSCs for the Z′-axis, at the high frequency end. This intercept point represents the series resistance, Rs, of the cells, arising from the FTO substrates and the external circuit wires.

Although there is a significant difference in the current-voltage characteristics of the solar cells, there are minimal differences in the electrochemical impedance spectroscopy data in terms of the resistance to the flow of charge and recombination losses as displayed in Nyquist and Bode plots.

## Conclusion

In conclusion, dye sensitized solar cells were fabricated with europium conjugated dendritic DL680 dye, 680-Eu-G5PAMAM, to study the effect of europium on the absorption characteristics of DL680 dye. The sensitizing dyes were first characterized using Absorption Spectroscopy, Emission Spectroscopy and Fluorescence lifetime, Fourier Transform Infrared measurements. They were also characterized using the Transmission electron microscope to obtain images and the respective sizes of the dye particles. The photovoltages characteristics of their respective dye sensitized solar cells were compared. The solar-to-electric conversion efficiency of DL680-Eu-G5PAMAM DSSC was 0.32% with a short circuit current of 1.66 mA/cm^2^, open circuit voltage of 0.46 V and a fill factor of 0.42 while solar-to-electric conversion efficiency of DL680 was 0.19% with a short circuit current of 1.84 mA/cm^2^, open circuit voltage of 0.35 V and a fill factor of 0.30. The enhancement of the efficiency is suggested to be due to the presence of the europium in the dendritic dye.

## Figures and Tables

**Table 1:** Current voltage characteristics of DL680-Eu-G5PAMAM and DL680 dye sensitized solar cells.

DSSC	V_max_	I_max_	V_oc_	I_sc_/A	FF	Efficiency (%)
DL680-Eu-G5PAMAM	0.28	1.13	0.46	1.66	0.42	0.32
DL680	0.19	1.01	0.35	1.84	0.30	0.19

**Table 2:** Fluorescence lifetime measurement of DL680 and DL680-Eu-G5PAMAM.

Sample	Lifetime (τ_1_) (ns)	Standard Deviation (σ)
DL680-Eu-G5PAMAM	2.11	0.0027
DL680	1.25	0.0025
